# Peer victimization and the association with hippocampal development and working memory in children with ADHD and typically-developing children

**DOI:** 10.1038/s41598-021-95582-7

**Published:** 2021-08-12

**Authors:** Alissa Papadopoulos, Diane Seguin, Susana Correa, Emma G. Duerden

**Affiliations:** 1 Applied Psychology, Faculty of Education, Western University, 1137 Western Rd, London, ON N6G 1G7 Canada; 2 Physiology and Pharmacology, Schulich School of Medicine and Dentistry, Western University, London, Canada; 3 Neuroscience, Schulich School of Medicine and Dentistry, Western University, London, Canada; 4 Psychiatry, Schulich School of Medicine and Dentistry, Western University, London, Canada

**Keywords:** Development of the nervous system, Neuroscience

## Abstract

The symptoms of hyperactivity-impulsivity and inattention displayed by children with ADHD put them at risk of experiencing peer victimization. Hippocampal maturation, may reduce a child’s vulnerability to the experience of peer victimization, as it has been associated with decreased ADHD symptomatology. Working memory is an important executive function in the formation and maintenance of social relationships, which is often impaired in ADHD. We aimed to evaluate the relationship between problem behaviours, peer victimization, hippocampal morphology, and working memory in children with and without ADHD. 218 typically-developing participants (50.5% male) and 232 participants diagnosed with ADHD (77.6% male) were recruited. The ADHD group was subdivided into inattentive (ADHD-I) or combined (ADHD-C) types. The Child Behavior Checklist measured problem behaviours and peer victimization. Children underwent Magnetic Resonance Imaging (MRI). Hippocampal subfield volumes were obtained using FreeSurfer. The Wechsler Intelligence Scale for Children-fifth edition measured working memory (WM). The ADHD-C group displayed significantly higher levels of problem behaviours and peer victimization (all, *p* < 0.001), compared to the other groups. Left Cornu Ammonis 3 (CA3) volume was a positive predictor of peer victimization (all, *p* < 0.013). Left CA3 volume was a positive predictor of WM and left Cornu Ammonis 4 (CA4) volume negatively predicted WM (all, *p* < 0.025). A cluster analysis revealed that children displaying symptoms of hyperactivity-impulsivity are the most at risk for peer victimization. Interventions focusing on minimizing peer victimization may aid in mitigating adverse downstream effects, and assist in promoting brain health and cognitive function.

## Introduction

Attention Deficit Hyperactivity Disorder (ADHD) affects approximately 5% of the child and youth population worldwide and is characterized by symptoms of hyperactivity-impulsivity and inattention^1,2^. ADHD is further characterized into three diagnostic subtypes, including ADHD hyperactive-impulsive type (ADHD-H), ADHD inattentive type (ADHD-I), and ADHD combined type (ADHD-C)^1,3^. ADHD-H is characterized by clinically elevated symptoms of hyperactivity-impulsivity^1^. ADHD-I is categorized by clinically elevated symptoms of inattention and children with ADHD-C display both clinically elevated hyperactivity-impulsivity and inattention symptoms^1^.

Children with ADHD have deficits in social skills and often act inappropriately in social situations, such as being disruptive and intrusive, which make maintaining healthy peer relationships difficult^4,5^. These social difficulties make children with ADHD susceptible to peer victimization and research has suggested that children with ADHD experience the highest rates of peer victimization when compared to children with other disabilities such as Autism Spectrum Disorder (ASD), Oppositional Defiant Disorder (ODD), intellectual disabilities, learning disabilities, and physical disabilities^4–6^. However, recent research has demonstrated that the levels of peer victimization experienced by children with ADHD vary according to their diagnosis subtype, with symptoms of hyperactivity-impulsivity centrally implicated as a factor that increases risk of experiencing higher levels of peer victimization^7^.Children with more severe hyperactivity-impulsivity symptoms also tend to exhibit more problem behaviours, such as rule breaking and externalized aggression, which can negatively influence the ability to form healthy peer relationships^4,5^. This suggests that children and youth with ADHD-H and ADHD-C are more susceptible to experiencing peer victimization than those with ADHD-I. Peer relationships play an important role in child development, and victimization by peers, including verbal, emotional, and physical abuse, can be a significant and potentially traumatizing stressor in a young person’s life^8,9^.

Children with ADHD differ in their brain structure compared to their typically-developing (TD) counterparts^10^. However, for many brain regions, including the hippocampus, how the structure differs between ADHD and TD children is unclear^11–15^. In some studies, enlarged hippocampal volumes are reported in children with ADHD compared to TD children^12^, some studies report decreased hippocampal volumes in children with ADHD compared to TD children^13,14^, and others report no differences^15^. However, few studies have taken diagnosis subtype into account when assessing hippocampal volume in ADHD. In a recent study with a large group of 880 children, Al-Amin et al. (2018)^16^ found that those diagnosed with ADHD-C had significantly smaller hippocampal volumes compared to a TD group, however hippocampal volumes were not significantly different between TD children and those with ADHD-I. In addition, they found that that larger hippocampal volumes negatively correlated with levels of ADHD symptomatology, a finding consistent with Plessen et al. (2006)^12^. As such, it has been suggested that hippocampal growth acts as a compensatory mechanism in response to the presence of ADHD symptoms^12^. Therefore, we hypothesized that in children with ADHD, those with larger hippocampal volumes might be at a decreased risk of experiencing the adverse effects of peer victimization and those that present with smaller hippocampal volumes might be more vulnerable, as they most likely display higher levels of ADHD symptomatology that more severely impact their ability to form peer relationships^7^.

The research on peer victimization and its association with hippocampal volume is scarce. In some studies, smaller hippocampal volumes were associated with higher levels of reported victimization in TD children, but further research is required to obtain a better understanding of this association^9,17^. Further, the association between peer victimization and hippocampal volume in the ADHD population and how this differs between ADHD subtypes has not been studied.

In addition to being at an increased risk of peer victimization due to their symptomatology, children with ADHD also display deficits in working memory (WM)^18,19^. Some studies have found that, in children, WM ability is  associated with social functioning^20^, and one study specifically observed WM deficits were related to poorer social functioningin children with ADHD^21^. The hippocampus is a key structure in WM and other executive functions, however the relationship between hippocampal volume and performance on tasks of executive function in children is not well defined^22–24^. In adult and aging populations, larger hippocampal volumes are associated with better performance on tasks of executive functioning, including WM^22–24^. However, this same relationship is not consistently observed in children, with some evidence suggesting that children with larger hippocampal volumes perform worse on tasks of memory^23^. The relationship between hippocampal volume and WM ability in the ADHD population remains largely unexplored.

In the current work, we examined the association amongst problem behaviours, peer victimization, hippocampal volume and WM function in a large heterogenous sample of children with ADHD and TD children. We addressed three main research questions: 1) Are problem behaviours and peer victimization predicted by ADHD diagnostic category? 2) Do hippocampal subfield volumes predict problem behaviours and peer victimization levels? 3) Do hippocampal subfield volumes predict WM ability? Our central hypothesis is that participants with ADHD will report higher levels of problem behaviours and peer victimization compared to TD children, and that hippocampal subregion volumes will significantly predict levels of peer victimization as well as WM ability.

## Methods

### Participants

Participants in this study ranged in age from 6.0 to 17.7 years. Data were collected by researchers at the Child Mind Institute and participants were recruited as part of the Healthy Brain Network initiative^25^. TD participants and those with a clinical diagnosis of ADHD were tested at three different sites: Rutgers University (RU), Citigroup Biomedical Imaging Center (CBIC), and Staten Island Diagnostic Research Center (SI). The research ethics boards at all respective institutions approved the study. Written informed consent was obtained from the participants’ parents and written assent was obtained from the participants. A breakdown of participant demographics can be found in Table 1.

**Table 1:** Characteristics of TD, ADHD-C and ADHD-I participants.

	TD n = 218	ADHD-C n = 108	ADHD-I n = 124
**Age, years, median [IQR]**	9.80 [7.90 to 12.72]	8.80 [7.26 to 11.24]	10.60 [8.58 to 13.05]
**Male, % (n)**	50.5 (110)	79.6 (86)	75.8 (94)
**MRI site, % (n)**^**a**^			
CBIC	14.2 (31)	25.0 (27)	27.0 (34)
RU	34.9 (76)	31.5 (34)	47.6 (59)
SI	26.1 (57)	16.7 (18)	5.60 (7)
Unknown	0.9 (2)	0 (0)	0 (0)
**BSMSS total, median [IQR]**	53.00 [46.00 to 61.00]	53.00 [44.88 to 61.00]	53.00 [45.00 to 59.50]
**SWAN, median [IQR]**			
Hyperactivity-Impulsivity	− 0.11 [− 1.42 to 0.22]	1.11 [0.67 to 1.67]	0.22 [0.00 to 0.67]
Inattention	− 0.06 [− 1.22 to 0.44]	1.11 [0.67 to 1.67]	1.22 [0.56 to 1.89]
**CBCL, median [IQR]**			
Aggressive behaviour	3.00 [0.00 to 5.00]	8.00 [4.25 to 13.00]	4.00 [1.00 to 8.00]
Rule breaking behaviour	1.00 [0.00 to 2.00]	3.00 [1.00 to 5.00]	2.00 [0.00 to 3.00]
Social problems	1.00 [0.00 to 3.00]	3.50 [1.00 to 6.00]	2.00 [0.00 to 3.75]
Withdrawn	1.00 [0.00 to 2.00]	1.00 [0.00 to 3.00]	1.00 [0.00 to 3.00]
Peer victimization	0.00 [0.00 to 0.00]	1.00 [0.00 to 2.00]	0.00 [0.00 to 1.00]
**WISC-V, median [IQR]**			
FSIQ	106.00 [95.00 to 113.00]	105.00 [91.75 to 113.00]	99.00 [88.00 to 109.00]
WMI	100.00 [91.00 to 112.00]	100.00 [91.00 to 110.00]	94.00 [88.00 to 107.00]
**Brain Volumes, Median [IQR]**			
TCV (mm^3^)	1,176,718.50 [1104748.00 to 1,268,853.75]	1,202,310.00 [1104571.00 to 1,260,378.00]	1,217,219.00 [1142320.00 to 1,334,728.00]
Left CA3 (mm^3^)	178.68 [162.48 to 199.21]	178.74 [165.64 to 203.56]	200.11 [182.77 to 216.74]
Left CA4 (mm^3^)	234.68 [215.67 to 255.58]	233.00 [218.29 to 252.00]	250.05 [230.20 to 268.30]

Clinical and demographic factors: IQR, interquartile range; CBIC, Citigroup Biomedical Imaging Center; RU, Rutgers University; SI, Staten Island Diagnostic Research Center; BSMSS, Barratt Simplified Measure of Social Status; SWAN, Strengths and Weaknesses Assessment of ADHD and Normal Behaviour; CBCL, Child Behaviour Checklist; TCV, total cerebral volume; Left CA3, Cornu Ammonis 3; WISC-V, Wechsler Intelligence Scale for Children-v; FSIQ, Full Scale IQ; WMI, Working Memory Index.

^a^MRI site used as covariate in aims 2 and 3.

In combination with clinical judgment, the computerized version of the Kiddie Schedule for Affective Disorders and Schizophrenia (K-SADS-COMP) was administered under the supervision of a licensed clinician to make diagnoses of ADHD, and to confirm the absence of ADHD diagnoses in TD participants^26^. Participants diagnosed with ADHD were further divided into subgroups, those diagnosed with ADHD-I and those diagnosed with ADHD-C. Participants with an ADHD-H diagnosed were excluded from this study due to a small sample size of only 15 participants having received this subtype diagnosis. All participants in the study were fluent in English, had a parent that was able to complete informant questionnaires, had an Intelligence Quotient (IQ) score of over 66, and were free of brain injury or disease. Participants were excluded if they presented with schizophrenia or bipolar disorder, psychosis, substance dependence, acute intoxication, a neurodegenerative disorder, or any other neurodevelopmental disorder. The study was conducted in accordance with the Declaration of Helsinki.

### Procedures

The data for this study was collected in 4 study visits. In the first visit, the WISC-V was administered by a trained psychometrist. The second visit consisted of the MRI protocol, including the T1-weighted sequence conducted by a trained MRI technician. During the scan participants watched two cartoon movies, the first movie was a short-film titled ‘The Present’ and the second was a 10-min clip from the full-length film ‘Despicable Me.’ Parent questionnaires were completed during the third visit and the K-SADS-COMP was completed during the fourth and final visit.

### Demographic measures

The Barratt Simplified Measure of Social Status (BSMSS) was used as a measure of socioeconomic status (SES) and was completed by a parent^27^. The BSMSS assesses level of education and occupation for both of a child’s parents, or from one parent if the child is from a single-parent household^27^. The scores were converted into a total score between 8 and 66 for each child, with a higher score indicative of a higher SES^27^.

### Psychological measures

To assess symptoms of hyperactivity-impulsivity and inattention, the Strengths and Weaknesses Assessment of ADHD and Normal Behaviour (SWAN) was completed by a parent^28^. To measure levels of problem behaviours in the participants, the Child Behaviour Checklist (CBCL) was administered to parents^29^. The subscales used from the CBCL in this study included the aggressive behaviour, rule breaking behaviour, social problems and withdrawn scales. In addition, peer victimization was measured using the CBCL subscale developed by McCloskey and Stuewig^30^. The peer victimization subscale contained four questions: “doesn’t get along with other kids”, “gets in many fights”, “gets teased a lot”, and “not liked by other children.” The Wechsler Intelligence Scale for Children-fifth edition (WISC-V) was used to obtain a Working Memory Index (WMI) in all participants, which included a digit span and a picture span task^31^.

### Magnetic resonance imaging

A Siemens 3 T Trio scanner was used at the Rutgers University site^25^. At the Staten Island Diagnostic Research Center, participants were scanned on a 1.5 T Siemens Avanto scanner and at Citigroup Biomedical Imaging Center participants were scanned on a Siemens 3 T Prisma^25^. To adjust for the differences in scanners, statistical models included Magnetic Resonance Imaging (MRI) site as a covariate.

High-resolution anatomical images were acquired using a 3D-MPRAGE pulse sequence with 192 T1-weighted, straight sagittal slices (1 mm thickness).

### Hippocampal segmentation

To obtain hippocampal subfield volumes from the structural images, FreeSurfer (http://surfer.nmr.mgh.harvard.edu) version 6.0. was used to segment and isolate hippocampal structures at high-resolution^32^. The hippocampus was segmented, bilaterally, into the hippocampal tail, subiculum, CA1, hippocampal fissure, presubiculum, parasubiculum, molecular layer, granule cell layers of the dentate gyrus (GCMLDG), CA3, CA4, fimbria, and the hippocampus-amygdala-transition-area (HATA). The total cerebral volumes were extracted using the Freesurfer pipeline.

### Statistical analysis

Statistical analyses were completed with the IBM SPSS Statistics software package (version 26, Statistical Package for the Social Sciences, IBM, Armonk, NY).

Our first aim was to examine problem behaviour and peer victimization in children with TD, ADHD-I, and ADHD-C. In five general linear models, we examined parent-reported problem behaviours and peer victimization (social problems, aggressive behaviour, rule breaking behaviour, withdrawal and peer victimization subscale raw scores; dependent variables) related to diagnostic group (TD, ADHD-I, ADHD-C; independent variables). Age, sex, SES (using the BSMSS), and study site were adjusted for in each model.

Our second aim was to examine problem behaviours and peer victimization and their relationships to hippocampal subfield volume*.* In four general linear models we examined peer victimization (subscale raw score; dependent variable), aggressive behaviour (subscale raw score; dependent variable), rule breaking behaviour (subscale raw score; dependent variable), and social problems (subscale raw score; dependent variable) in relation to hippocampal subfield volumes (right and left hippocampal tail, subiculum, CA1, hippocampal fissure, presubiculum, parasubiculum, molecular layer, GCMLDG, CA3, CA4, fimbria and HATA; independent variables), while adjusting for age, sex, SES (i.e., BSMSS), diagnostic group, total cerebral volume (TCV), and MRI site.

Our third aim was to examine the association of hippocampal subfield volumes and working memory ability*.* A general linear model was used to examine the relationship between the left and right hippocampal subfield volumes (hippocampal tail, subiculum, CA1, hippocampal fissure, presubiculum, parasubiculum, molecular layer, GCMLDG, CA3, CA4, fimbria and HATA; independent variables), and WM (WISC-V: WMI standardized score; dependent variable). We adjusted for age, sex, SES (i.e., BSMSS), diagnostic group, TCV, and MRI site.

## Results

### Participant demographics

A total of 218 TD participants (median age = 9.80 years; interquartile range[IQR] = 7.90–12.72; 50.5% male) and 232 participants diagnosed with ADHD (median age = 9.82 years; 77.6% male) were recruited. Of the 232 participants diagnosed with ADHD, 108 participants had the ADHD-C subtype (median age = 8.80 years; IQR = 7.26–11.24 years; 79.6% male) and 124 participants had the ADHD-I subtype (median age = 10.60 years; IQR = 8.58–13.05 years; 75.8% male). Participant characteristics can be found in Table 1.

### Problem behaviours, peer victimization and diagnostic group

In our first aim, we examined the relationship between problem behaviours from the CBCL, including social problems, rule breaking behaviour, aggressive behaviour, and withdrawn, and diagnostic group (TD, ADHD-I, and ADHD-C). We also examined how the experience of peer victimization differs by diagnostic group (TD, ADHD-I, and ADHD-C). Age, sex, SES, and study site were adjusted for in all the models. In the problem behaviours models 433 participants were included in analysis, 204 TD children, 123 children with ADHD-I, and 106 children with ADHD-C. In the peer victimization model, a total of 431 participants were included in the analysis, 203 TD children, 123 children with ADHD-I, and 105 children with ADHD-C. 

Compared to the TD group, children from both the ADHD-C (B = 5.42, 95% CI = 4.26–6.58, *p* < 0.001) and ADHD-I (B = 2.41, 95% CI = 1.32–3.51, *p* < 0.001) groups had significantly higher scores on the aggressive behaviour subscale. ADHD-C participants also had significantly higher scores than the TD participants on the rule breaking behaviour (B = 1.95, 95% CI = 1.39–2.50, *p* < 0.001) and social problems (B = 2.16, 95% CI = 1.53–2.78, *p* < 0.001) subscales. No significant differences were found between the TD and ADHD-C participants on the withdrawn subscale (B = 0.51, 95% CI = 0.03–0.99, *p* = 0.038). Nor were significant differences found between the TD and ADHD-I participants on the rule breaking behaviour (B = 0.62, 95% CI = 0.10–1.15,* p* = 0.021), social problems (B = 0.75, 95% CI = 0.16–1.33, *p* = 0.013), and withdrawn (B = 0.49, 95% CI = 0.04–0.94, *p* = 0.033) subscales. In comparing children with ADHD-C to children with ADHD-I, we found that those with ADHD-C had significantly higher scores on aggressive behaviour, rule breaking behaviour, and social problems (all, *p* < 0.001).

**Figure 1:**
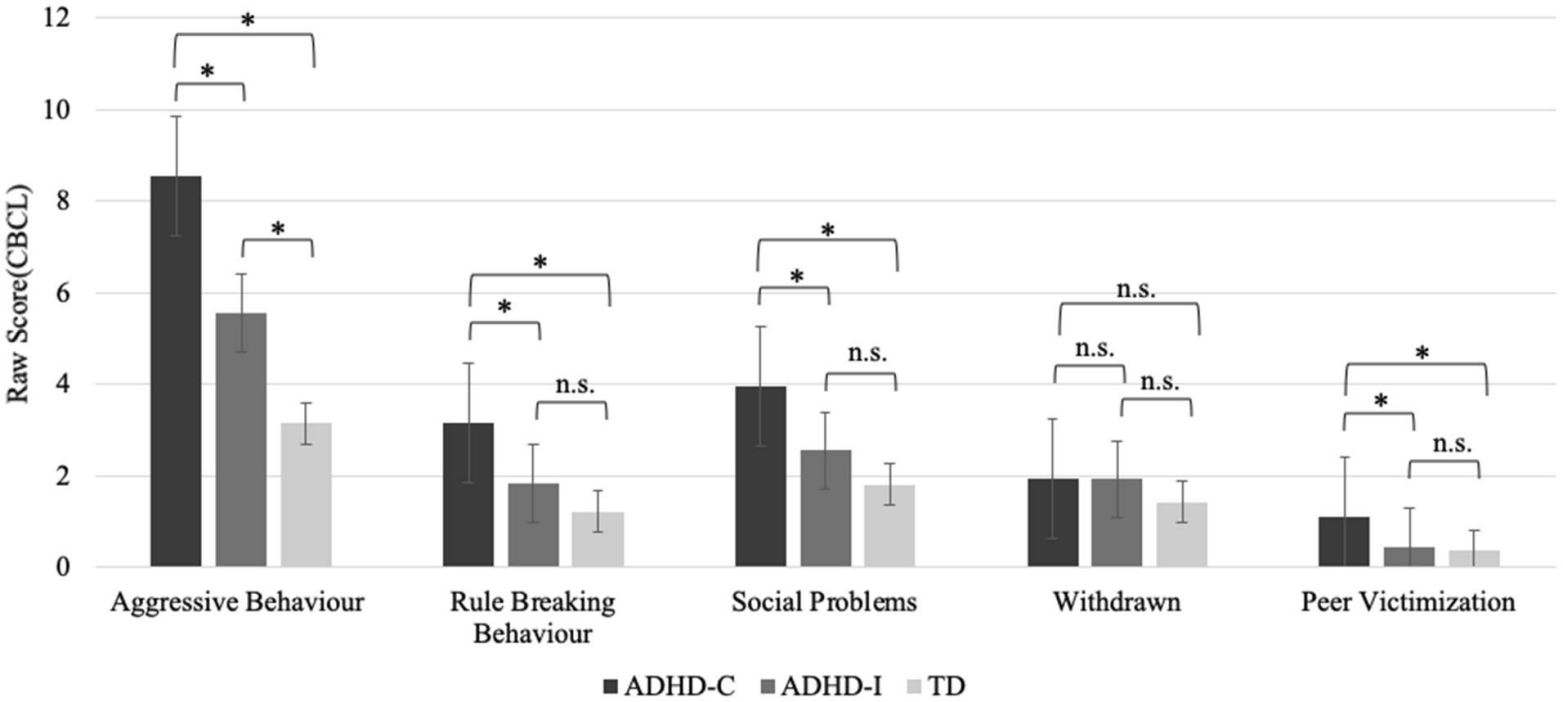
Problem behaviours and peer victimization in diagnostic groups. CBCL subscale scores for children from the ADHD-C, ADHD-I, and TD groups. Children from the ADHD-C group had significantly higher aggressive behaviour, rule breaking behaviour, social problems, and peer victimization scores compared to the TD group. The ADHD-C group also had significantly higher aggressive behaviour, rule breaking behaviour, social problems and peer victimization scores compared to the ADHD-I group. Children and adolescents in the ADHD-I group had significantly higher aggressive behaviour scores than the TD group. None of the groups significantly differed on the withdrawn scores. Scores represent the estimated marginal means, adjusted for age, sex, study site, and SES. P values are Bonferroni corrected (pairwise) for multiple comparisons. Error bars reflect standard error. ^*^p < 0.001.

When examining the peer victimization subscale, children with ADHD-C had significantly higher levels of peer victimization compared TD children (B = 0.73, 95% CI = 0.47–0.98, *p* < 0.001), but there was no significant difference found for peer victimization between the ADHD-I and TD groups (B = 0.09, 95% CI = − 0.16–0.33, *p* = 0.483). In addition, the children with ADHD-C had significantly higher peer victimization scores than the children with ADHD-I (*p* < 0.001). Refer to Fig. 1 for a summary of the results.

### Problem behaviours, peer victimization and hippocampal subfield volumes

In our second aim, we examined whether hippocampal subfield volumes could predict problem behaviours and levels of peer victimization. The problem behaviours examined in this aim included the aggressive behaviour, rule breaking behaviour, and social problems subscales, adjusting for age, sex, SES, MRI site, diagnostic group, and TCV. The analysis was run with a total of 282 participants, including 136 TD children, 83 children with ADHD-I, and 63 children with ADHD-C.

Left CA3 volume was positively associated with peer victimization (B = 0.019, 95% CI = 0.005–0.034, *p* = 0.010). Left CA3 volume showed a positive but not-significant association with rule breaking behaviour (B = 0.034, 95% CI = 0.003–0.066, *p* = 0.032), aggressive behaviour (B = 0.054, 95% CI = − 0.010–0.119, *p* = 0.097), and social problems (B = 0.029, 95% CI = − 0.006–0.064, *p* = 0.107).

We subsequently examined the interaction between left CA3 volume and diagnostic group (left CA3 volume x diagnostic group) in the peer victimization model. We found that left CA3 volume was significantly and positively associated with peer victimization in in TD children (B = 0.018, 95% CI = 0.003–0.33, *p* = 0.017), children with ADHD-C (B = 0.022, 95% CI = 0.007–0.036, *p* = 0.004), and children with ADHD-I (B = 0.017, 95% CI = 0.003–0.032, *p* = 0.022).

### Hippocampal subfield volumes and working memory ability

This analysis included a total of 243 participants, including 109 TD children, 76 children with ADHD-I, and 58 children with ADHD-C. When adjusting for age, sex, SES, MRI site, TCV, and hippocampal subfield volumes the ADHD-I group had significantly poorer WMI scores than the TD group (B = − 5.49, 95% CI = − 9.75 to − 1.23, *p* = 0.011), but the ADHD-C group did not significantly differ from the TD group (B = − 4.31, 95% CI = − 8.95–0.33, *p* = 0.069).

We examined the relationship between hippocampal subfield volumes and WMI. Left CA3 volume was found to be significantly and positively associated with WMI (B = 0.233, 95% CI = 0.042–0.424, *p* = 0.017). Statistical interactions were tested in the WMI model between left CA3 volume and diagnostic group (left CA3 x diagnostic group). Left CA3 volume was significantly and positively associated with WMI in TD children (B = 0.249, 95% CI = 0.59–0.440, *p* = 0.010), children with ADHD-C (B = 0.230, 95% CI = 0.038–0.422, *p* = 0.019), and children with ADHD-I (B = 0.220, 95% CI = 0.30–0.411, *p* = 0.023).

Left CA4 volume was found to be significantly and negatively associated with WMI (B = − 0.626, 95% CI = − 1.14 to − 0.107, *p* = 0.018). Statistical interactions were also tested in the WMI model between left CA4 and diagnostic group (left CA4 x diagnostic group). Left CA4 volume was significantly and negatively associated with WM in TD children (B = − 0.608, 95% CI = − 1.128 to − 0.089, *p* = 0.022), children with ADHD-C (B = − 0.625, 95% CI = − 1.141 to − 0.108, *p* = 0.018), and children with ADHD-I (B = − 0.632, 95% CI = − 1.151 to − 0.113, *p* = 0.017).

As anxiety and depression may also be important predictors of hippocampal volumes in children with ADHD, we further examined the association amongst WMI and brain development in a post-hoc analysis using the anxious/depressed subscale of the CBCL as a covariate. Scores on the anxious/depressed scale were not significantly related to WMI (B = − 0.489, *p* = 0.080). In this post-hoc model, the associations between WMI and left CA3 (B = 0.245, *p* = 0.012) and left CA4 (B = − 0.622, *p* = 0.018) remained significant.

### Post-hoc cluster analysis

To examine the associations amongst diagnosis, hippocampal volumes, WM as well as ADHD symptomatology, a K-means cluster analysis was performed with 245 participants: 111 TD, 58 ADHD-C, and 76 ADHD-I. The analysis included variables of brain morphology, behaviour, and cognition (Z-scored). The model included, left CA3 volume (*p* = 0.018), peer victimization score (*p* < 0.001), WMI (*p* < 0.001), and the hyperactivity-impulsivity and inattention subscales from the SWAN (both *p* < 0.001). Refer to Table 2 for a summary of the clusters.

**Table 2:** Three-cluster model participant demographics.

	Cluster 1 n = 41	Cluster 2 n = 54	Cluster 3 n = 150	*p* value
Left CA3 (mm^3^), Median [IQR]	180.00 [155.82 to 196.24]	183.01 [169.90 to 207.00]	191.08 [172.81 to 211.52]	0.018
WMI median [IQR]	107 [98.50 to 122.00]	100 [88.00 to 110.00]	97 [88.00 to 104.00]	< 0.001
Peer victimization median [IQR]	0 [0.00 to 0.00]	2 [2.00 to 3.25]	0 [0.00 to 0.00]	< 0.001
**SWAN**				
Hyperactivity-impulsivity median [IQR]	− 1.22 [− 2.78 to − 0.56]	0.72 [0.22 to 1.44]	0.22 [0.00 to 0.78]	< 0.001
Inattention median [IQR]	− 1.56 [− 2.22 to − 0.78]	1 [0.22 to 1.67]	0.78 [0.11 to 1.44]	< 0.001

Cluster 1 included a total of 41 participants and is characterized as children with small left CA3 volumes, high WMI, low peer victimization scores, and low hyperactivity-impulsivity and inattention symptomatology scores. This group is made up of 40 TD participants, and 1 participant from the ADHD-I group. All but 3 participants in this cluster scored 0.5 SD below the mean on the peer victimization scale. In this cluster 56% of the participants had left CA3 volumes that were below the average and 75% of the participants had higher average WMI. In regard to symptoms of hyperactivity-impulsivity and inattention, 39% of the participants in cluster 1 scored 1 SD below the mean and 34% scored below 2 SD below the mean.

Cluster 2 included a total of 54 participants. Participants in this group have average left CA3 volumes, average WMI, high peer victimization scores, and high hyperactivity-impulsivity and inattention symptomatology. This group is made up of 19 TD participants, 24 ADHD-C participants and 11 ADHD-I participants. All participants in this cluster were above average in peer victimization scores, with approximately 41% of the cluster at least 2 SD above the mean, and 56% above 1 SD. In this cluster 54% of participants had left CA3 volumes that were below average and 20% of participants had a WMI at least 1 SD below the average. In regard to symptomatology, 20% of the participants had hyperactivity-impulsivity scores at least 1 SD above the mean and 28% had inattention scores at least 1 SD above the mean.

Cluster 3 included a total of 150 participants and can be described as participants with large left CA3 volumes, low WMI, low peer victimization scores, and average hyperactivity-impulsivity and inattention symptomatology. This cluster is made up of 52 TD participants, 34 ADHD-C participants, and 64 participants from the ADHD-I group. In cluster 3, approximately 79% of the participants had peer victimization scores that were 0.5 SD below the mean, A total of 60% of the participants had left CA3 volumes that were above the mean, with 21% being least 1 SD above the average. Approximately 30% of this cluster had WMI above the mean, and most participants in this cluster were above average in hyperactivity-impulsivity and inattention symptomatology, 57% and 60% respectively.

**Figure 2:**
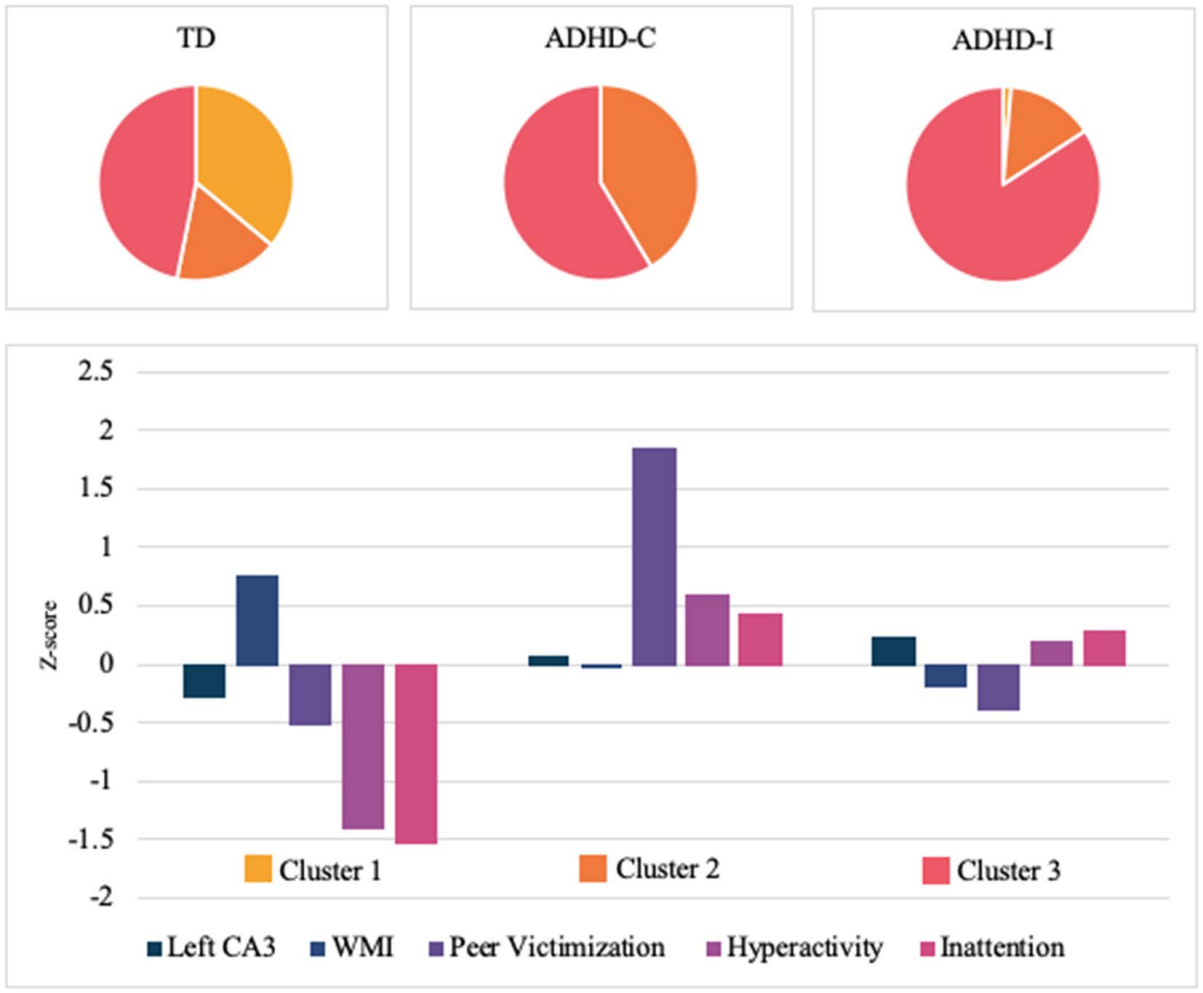
K-means clustering based on peer victimization, left CA3, WMI, hyperactivity-Impulsivity and inattention. The three-cluster model is depicted above. Cluster 1 is characterized by small left CA3 volume, high WMI, low peer victimization, and low hyperactivity-impulsivity and inattention. Cluster 1 is almost entirely made up of TD participants. Cluster 2 is characterized as average left CA3 volumes, average WMI, high peer victimization, and high hyperactivity-impulsivity and inattention. Of the participants in cluster 2, 44% are from the ADHD-C group. Cluster 3 is characterized as large left CA3 volume, low WMI, low peer victimization, and average hyperactivity-impulsivity and inattention. Cluster 3 is approximately 43% ADHD-I participants, 35% TD participants, and 22% ADHD-C participants.

When examining cluster membership by diagnostic groups, we found 36% of the total TD sample (n = 111) was grouped into cluster 1, 17% were grouped into cluster 2, and 47% were grouped into cluster 3. Of the 58 total ADHD-C participants, 41% were grouped into cluster 2, and 59% were grouped into cluster 3. Of the 76 total ADHD-I participants, 1% belonged to cluster 1, 14% were grouped into cluster 2, and 84% were grouped into cluster 3. Refer to Fig. 2 for a summary of the cluster analysis.

A one-way ANOVA was conducted to examine the differences between the clusters on all the variables. Significant between-group differences were found for all the variables: left CA3 volume (F(2, 242) = 4.07, *p* = 0.018), WMI (F(2,242) = 15.44, *p* < 0.001), peer victimization (F(2,242) = 364.86, *p* < 0.001), hyperactivity-impulsivity (F(2,242) = 103.63, *p* < 0.001), and inattention (F(2,242) = 112.24, *p* < 0.001). Post hoc multiple comparison using Bonferroni correction revealed the differences between the clusters. Significant differences were found between Cluster 1 and Cluster 2 on WMI, peer victimization, hyperactivity-impulsivity, and inattention (all *p* < 0.001). Significant differences were found between Cluster 1 and 3 on Left CA3 volume (*p* = 0.015), WMI, hyperactivity-impulsivity, and inattention (all *p* < 0.001). Significant differences were found between Cluster 2 and 3 on peer victimization (*p* < 0.001) and hyperactivity-impulsivity (*p* = 0.001).

## Discussion

The presence of problem behaviours, levels of peer victimization, hippocampal subfield morphology, and working memory were assessed in a large heterogenous sample of children further divided by diagnostic group: TD, ADHD-C and ADHD-I. We found that problem behaviours and levels of peer victimization differed between TD, ADHD-C, and ADHD-I groups. We also report that hippocampal subfield volumes predict the presence of peer victimization levels in children with ADHD and TD children. Lastly, hippocampal subfield volumes were associated with working memory ability in children with ADHD and TD children. Peer victimization was highest in children that displayed elevated levels of hyperactivity-impulsivity. Findings suggest that children with ADHD-C who display elevated levels of hyperactivity-impulsivity may be at greater risk for peer victimization.

### Problem behaviours, peer victimization and diagnostic group

As hypothesized, children in the ADHD diagnostic groups had elevated levels problem behaviours and peer victimization compared to TD children. Parents of children with ADHD-C reported higher levels of aggressive behaviour, rule breaking behaviour, social problems, and higher levels of experiencing peer victimization in their children compared to parental reports for TD children and those with ADHD-I. The children with ADHD-I only differed from the TD children when comparing levels of aggressive behaviour; with significantly higher reported levels in the ADHD-I group. No significant differences were found in levels of withdrawal between any of the diagnostic groups.

Children with ADHD are typically not well-liked by peers and can often experience social rejection and victimization^5^. Children with ADHD who display more severe levels of externalizing behaviours have been observed to be the most at-risk pediatric population to experience peer victimization^5,6^. Investigation into how peer victimization differs amongst the diagnostic subtypes of ADHD is sparse. Recent findings into the symptomatology of ADHD in relation to peer victimization suggest that more severe symptoms of hyperactivity-impulsivity are associated with higher rates of peer victimization among children^7^. Not only are hyperactive-impulsive symptoms related to experiencing more victimization, it has also been demonstrated that individuals with these symptoms are also more likely to be perpetrators of bullying themselves^7^. This notion is consistent with the findings from the present study. Although the peer victimization measure in the present study only accounted for experiencing victimization, the significantly elevated aggressive behaviour, rule breaking behaviour, and social problems scores in the ADHD-C group suggest that this group might also be perpetrators of victimization and fit the profile of a bully-victim^7^. As the ADHD-I group has levels of hyperactivity-impulsivity that are comparable to the TD group, it follows that they are victimized by their peers at rates similar to the TD group. Although the children with ADHD-I also had significantly elevated aggression scores compared to TD children, the lack of hyperactivity-impulsivity symptoms combined with low rates of peer victimization suggest that this group might fit into a different category than the ADHD-C group; that is, one of aggressive children and adolescents that are accepted by their peers^33^.

Although significant differences were found between the diagnostic subtypes in peer victimization and problem behaviours, some research has highlighted instability and unreliability in the ADHD diagnostic subtypes, especially as children age^34,35^. In the present study, the children in the ADHD-C group were younger than the group diagnosed with ADHD-I; however all models were adjusted for age. Age was not significantly associated with peer victimization, aggressive behaviour or rule breaking behaviour. However, younger age was associated greater social problems, and older age was associated with withdrawn behaviours. In the present study, we did not examine the associations of problem behaviours in younger versus older children. It would be a worthwhile avenue of future exploration to investigate problem behaviours and peer victimization between the ADHD subtypes in  specific age groups when these behaviours may be more likely to occur.

Overall, experiencing peer victimization may be traumatizing, with a tremendous impact on later psychological development^36^. It is crucial that future research focuses on examining the impact of different types of victimization on child and adolescent psychological health. Determining how different types of victimization effect children can inform intervention strategies and can be useful for informing school-based interventions.

### Hippocampal subfield volume, problem behaviours and peer victimization

Research suggests children with smaller hippocampal volumes are usually observed to display more severe ADHD symptomatology, and as a result may be more vulnerable to the adverse effects of peer victimization^7,12^. However, contrary to our hypothesis, in the current study larger left CA3 volumes were associated with higher parent-reported levels of peer victimization in children with ADHD-C, ADHD-I and TD children. Our peer victimization scale measured victimization in the past 6 months, however it is possible, and perhaps likely that the children who experienced peer victimization in the past 6 months, had been experiencing victimization over a longer period of time. The CA3 region is a particularly stress-sensitive area and has previously been associated with other forms of childhood adversity^8,37^. Typically, smaller hippocampal volumes have been associated with a greater incidence of trauma and adversity in TD children^37–39^. However, this association has been found to be different in children with mental health and neurodevelopmental disorders^40–42^. Some models suggest that chronically elevated levels of stress during childhood may cause reduced stress responsivity, which in turn may mitigate the neurotoxic effects glucocorticoids (GCs) typically have in atrophying the neurons of the hippocampus^39,41,43^. This cascade of events results in larger hippocampal volumes, as found in our study.

In addition, research conducted with pediatric post-traumatic stress disorder (PTSD) populations has provided evidence that hippocampal volumes are enlarged in PTSD when compared to  matched non-traumatized groups, suggesting that anxiety and stress may be associated with increased growth of the hippocampus^42^. A recent study found larger subiculum, presubiculum, and CA1 volumes to be associated with childhood trauma in bipolar children^41^. Children with ASD have also been found to have enlarged hippocampal volumes compared to TD children^40^ and larger hippocampal volumes in children with ADHD have also been observed^12^.

Future research in this area could focus on examining if and how the relationship between hippocampal volume and peer victimization differs according to cortisol levels. In addition, age and gender should be directly examined. To maintain the large sample size in the current study we were not able to split the data into subgroups; however, these factors were used as covariates. Age was not a significant covariate in the model relating hippocampal volumes and peer victimization. In addition, future research is warranted to elucidate the relationships between ADHD subtype, symptomatology and peer victimization to hippocampal volume, specifically including the ADHD-H subtype.

### Hippocampal subfield volume and working memory

The children with ADHD-I had significantly lower scores on the WMI when compared to TD children. The ADHD-C group did not significantly differ from the ADHD-I group nor the TD group on WMI scores. WM deficits are often seen in children with an ADHD diagnosis^18,19^. However, there is a lack of consensus on how the different ADHD subtypes differ in respect to WM ability, and cognitive ability in general. Some studies report no differences between high and low inattention symptoms and WM scores^44^. Other studies have found inattention to be a significant predictor of WM ability in adolescents with ADHD^45^. As children with ADHD-C also have clinically elevated symptoms of inattention, in turn it is unclear why the children with ADHD-C did not display WM deficits when compared to the TD children in our study.

Larger left CA3 volumes and smaller left CA4 volumes were significantly predictive of higher WMI scores in all of the diagnostic groups. CA3 and CA4 are both key hippocampal subfields that are involved in WM^46^. Larger volumes predict better cognitive function in adult samples^22–24^. Yet, findings in children and adolescents provide inconsistent results to support this view. In a meta-analysis by Van Petten^23^ multiple studies examining hippocampal volume and memory performance in child and adolescent populations found negative correlations between volume and memory abilities. This suggests that the positive relationship between cognitive ability and hippocampal brain volume may increase with age^23^. It has been found more recently that as children age, smaller hippocampal volumes are associated with superior memory abilities^47–49^. Previous studies examined whole hippocampal volumes. Future studies should investigate the relationship between hippocampal subfield volumes and WM ability in ADHD populations.

Anxiety, depression, and other common disorders that co-occur with ADHD can also impact WM performance^50,51^. However, some studies have suggested that children with ADHD and comorbid disorders do not perform signficiantly differently on tasks of executive functioning, such as WM, from children with an ADHD diagnosis alone^50^. A post-hoc analysis was conducted including the anxious/depressed subscale as a covariate in the WMI model. We found that the anxious/depressed scale was not significantly related to WMI and that left CA3 and left CA4 remained significant predictors of WMI, however future research should examine this further.

### ADHD subtypes, hippocampal volumes, working memory and peer victimization

A three-cluster K-means model was used to characterize the present study population. Cluster 1 included 40 TD children and 1 child with ADHD-I who have low levels of inattentive and hyperactive-impulsive symptomatology, good WM ability, and low peer victimization. Cluster 2 included 19 TD children, 24 children with ADHD-C and 11 children with ADHD-I who have significantly elevated levels of peer victimization and hyperactivity-impulsivity when compared to the two other clusters. Cluster 3 included 52 TD children, 34 children with ADHD-C and 64 children with ADHD-I who have significantly enlarged left CA3 volumes and significantly higher hyperactivity-impulsivity and WMI scores compared to children in Cluster 1.

The results of the cluster analysis suggest that ADHD symptomatology, especially hyperactivity-impulsivity, is related to increased levels of peer victimization. Our model also suggests that left CA3 volumes are associated with ADHD symptomatology. In both Clusters 2 and 3, symptoms of hyperactivity-impulsivity and inattention are significantly elevated and, in both groups, left CA3 volumes are enlarged compared to Cluster 1, although only significantly enlarged in cluster 3. Similarities can be seen between our model and the inverse correlation Plessen et al.^12^ observed between CA3 volume and symptoms of inattention in children with ADHD. In our model children in cluster 3 have the most prominent enlargement of left CA3 volumes, but milder symptoms of inattention than children in cluster 2. This suggests the possibility that enlarged left CA3 volumes are representative of a compensatory mechanism by which the hippocampus hypertrophies in response to the presence of ADHD symptoms and results in less severe symptomatology^12^. The relationship between symptoms of inattention in children with ADHD and hippocampal volume, specifically in the CA3 subfield should be further explored. Our results suggest that deep phenotyping of brain morphology, cognition, and behaviour can identify subtle differences in ADHD subtypes.

A limitation of the current study is our use of a parent-report questionnaire, the CBCL, as a measure of problem behaviour and peer victimization. Children have many experiences without their parents present, such as in school, recreational activities, etc., thus child and teacher reports may be able to provide additional information that cannot be captured by parent-report alone. However, only a limited subset of children had self- and teacher- reports available. This is an important area of future research. Another limitation of the current study was the absence of the ADHD-H. We only had access to data for 15 participants diagnosed with ADHD-H (16.5% of the total ADHD sample), which was not a large enough sample for valid analyses. In children and adolescents ranging from 3 to 18 years of age, ADHD-I is the most common ADHD subtype with a prevalence of between 2.2 and 5.7%, the prevalence of ADHD-C ranges from 1.1 to 2.4%, and the prevalence of ADHD-H ranges from 1.1 to 4.9%^52^. As children age, an ADHD-H diagnosis becomes less common and could be the reason we only had access to the data of very few children with this subtype. Another limitation is that different scanners with different strengths were used at the three MRI sites. However, a majority of the participants were scanned at CBIC and RU, which both had 3 T scanners. The medication status of the children and adolescents in the study sample are unknown and this is a potential confound for behavioural reports, hippocampal volumes and WM performance.

## Data Availability

The datasets generated and/or analysed during the current study are available in the Child Mind Institute Healthy Brain Network repository, http://fcon_1000.projects.nitrc.org/indi/cmi_healthy_brain_network/.
